# Generation and Characterization of CYP2E1-Overexpressing HepG2 Cells to Study the Role of CYP2E1 in Hepatic Hypoxia-Reoxygenation Injury

**DOI:** 10.3390/ijms24098121

**Published:** 2023-05-01

**Authors:** Nouf Alwadei, Mamunur Rashid, Devaraj Venkatapura Chandrashekar, Simin Rahighi, Jennifer Totonchy, Ajay Sharma, Reza Mehvar

**Affiliations:** Department of Biomedical and Pharmaceutical Sciences, School of Pharmacy, Chapman University, Irvine, CA 92618, USA; alwadei@chapman.edu (N.A.); mamunrashid087@gmail.com (M.R.);

**Keywords:** HepG2 cells, hepatic ischemia-reperfusion, hypoxia-reoxygenation, cytochrome P450, CYP2E1, reactive oxygen species, cell death

## Abstract

The mechanisms of hepatic ischemia/reperfusion (I/R) injury, which occurs during liver transplantation or surgery, are poorly understood. The purpose of the current study was to generate and characterize a HepG2 cell line with a stable overexpression of CYP2E1 to investigate the role of the enzyme in hypoxia/reperfusion (H/R) injury in an ex vivo setting. GFP-tagged CYP2E1 and control clones were developed, and their gene expression and protein levels of GFP and CYP2E1 were determined using RT-PCR and ELISA/Western blot analysis, respectively. Additionally, the CYP2E1 catalytic activity was determined by UPLC-MS/MS analysis of 6-hydroxychlorzoxazone formed from the chlorzoxazone substrate. The CYP2E1 and control clones were subjected to hypoxia (10 h) and reoxygenation (0.5 h), and cell death and reactive oxygen species (ROS) generation were quantitated using LDH and flow cytometry, respectively. Compared with the control clone, the selected CYP2E1 clone showed a 720-fold increase in CYP2E1 expression and a prominent band in the western blot analysis, which was associated with a 150-fold increase in CYP2E1 catalytic activity. The CYP2E1 clone produced 2.3-fold more ROS and 1.9-fold more cell death in the H/R model. It is concluded that the constitutive CYP2E1 in the liver may play a detrimental role in hepatic I/R injury.

## 1. Introduction

Hepatic ischemia/reperfusion (I/R) injury due to vascular occlusion is the major cause of graft dysfunction and failure during both liver transplantation and liver surgery [1,2]. There are two phases in the injury process. The acute injury phase (early phase), which occurs within one to six hours of reperfusion, is marked by the activation of Kupffer cells, pro-inflammatory cytokine release, and reactive oxygen species (ROS) generation [3,4]. Following the acute phase is the subacute phase (late phase), which is characterized by neutrophil infiltration, and an increased formation of inflammatory mediators [4,5].

Several mechanisms contribute to the overall hepatic damage caused by I/R injury [1,6,7,8,9,10]. As a result of the interruption in blood supply and lack of oxygen, hepatocytes, owing to their high metabolic demand, suffer an ischemic injury, which is exacerbated by the reinstatement of the oxygenated blood. Although the exact mechanisms remain unclear, ROS are a key factor in this multistep damage process [11]. As a result of ischemia, Kupffer cells are activated, and ROS are released, causing the peroxidation of proteins, lipids, and DNA. Although there are no specific treatments for hepatic I/R injury, several approaches have been used to mitigate the injury. These approaches include Kupffer cell activation inhibitors, various antioxidants, and antibodies against leukocyte/endothelial cell adhesion molecules [4,11]. Among these approaches, investigators have proposed that ROS could be a promising therapeutic target due to its important role in this damage [11]. 

It has been established that several enzyme systems are involved in ROS production during I/R injury to various organs, including xanthine oxidase, NADPH oxidase, and (uncoupled) mitochondrial electron transport [11]. More recently, cytochrome P450 (P450) enzymes have been proposed as generators of ROS [12,13], and I/R injury in the heart [14], kidney [15], and brain [16]. Cytochrome P450 enzymes are hemeproteins that are responsible for the phase I metabolism of xenobiotics, carcinogens, toxins, and endogenous compounds [17,18,19]. P450 enzymes generate ROS by the uncoupling of the P450 catalytic cycle [12,20,21]. Additionally, the degradation of P450 enzymes release heme and iron, which may contribute to ROS generation through the Fenton reaction [22]. The P450-induced ROS generation is likely highest in the liver, compared with the other organs. This is because the liver has the highest P450 content, and P450 enzymes are the major source of heme and iron in the liver [23]. Therefore, P450 enzymes may contribute to the hepatic I/R injury by ROS generation through the uncoupling and/or release of iron due to their degradation. Indeed, it has been reported [24,25] that hepatic I/R causes the degradation of P450, which may be contributing to the I/R injury. 

Cytochrome P450 2E1 (CYP2E1) is one of the important P450 enzymes, which plays an important role in the metabolism of a number of drugs and in the generation of reactive metabolites from ethanol and environmental chemicals [26]. In addition to its physiological role in degradation of some drugs, increased levels of CYP2E1 contributes to several pathophysiological conditions such as diabetes, obesity, cancer, and liver diseases [26]. Among P450 enzymes, CYP2E1 is unique because it is known to have a high oxidase activity in the absence of substrates, causing a significant production of ROS [26,27]. Additionally, CYP2E1 is the most unstable of all liver microsomal P450 isoforms, with a rapid turnover half-life of 4–7 h [27]. Therefore, it is crucial to determine the potential role of CYP2E1 in hepatic I/R injury. In the current study, we developed and characterized a CYP2E1-overexpressing HepG2 cell line for use in an ex vivo hypoxia/reoxygenation (H/R) model to investigate the role of CYP2E1 in ROS generation and H/R injury. 

## 2. Results

### 2.1. Characterization of CYP2E1-Overexpressing HepG2 Cells

**Figure 1:**
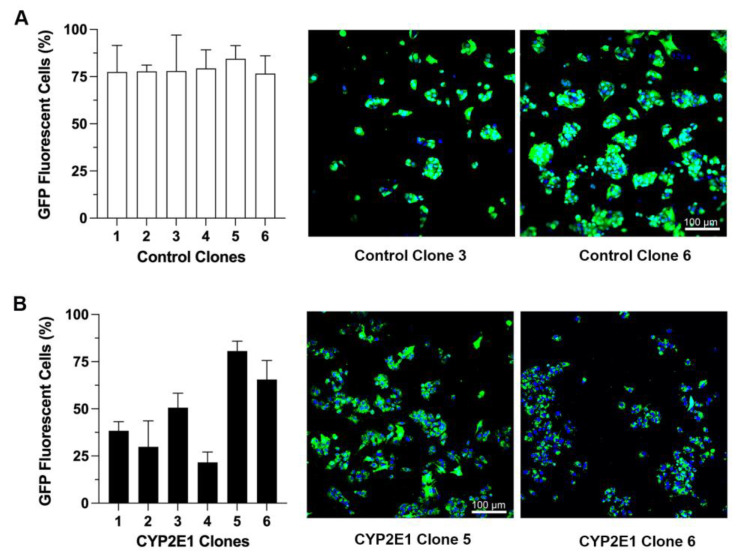
The percentages of cells showing GFP fluorescence for the clones transfected with the control (GFP only) (**A**) or CYP2E1 (GFP plus CYP2E1) (**B**) plasmids. The columns and bars, respectively, represent the mean and standard deviations of the three measurements. The confocal fluorescence images for two clones from each group are also shown (green: GFP; blue: DAPI for nuclear staining).

After transfecting HepG2 cells with the CYP2E1 or control plasmids and subsequent G418 selection, six stable clones from each group were selected, isolated, and grown to a large scale. We used the GFP expression-associated fluorescence as the initial marker to monitor and confirm stable transfection. Figure 1 demonstrates the percentages of cells showing GFP expression, along with representative confocal fluorescence images of two clones each, for the clones transfected with the control (GFP only) (Figure 1A), or the CYP2E1 (GFP and CYP2E1) (Figure 1B) plasmids. The percentages of cells showing GFP expression ranged from 76.6% (clone 6) to 84.4% (clone 5) for the control clones (Figure 1A), and 21.6% (clone 4) to 84.4% (clone 5) for the CYP2E1 clones (Figure 1B).

**Figure 2:**
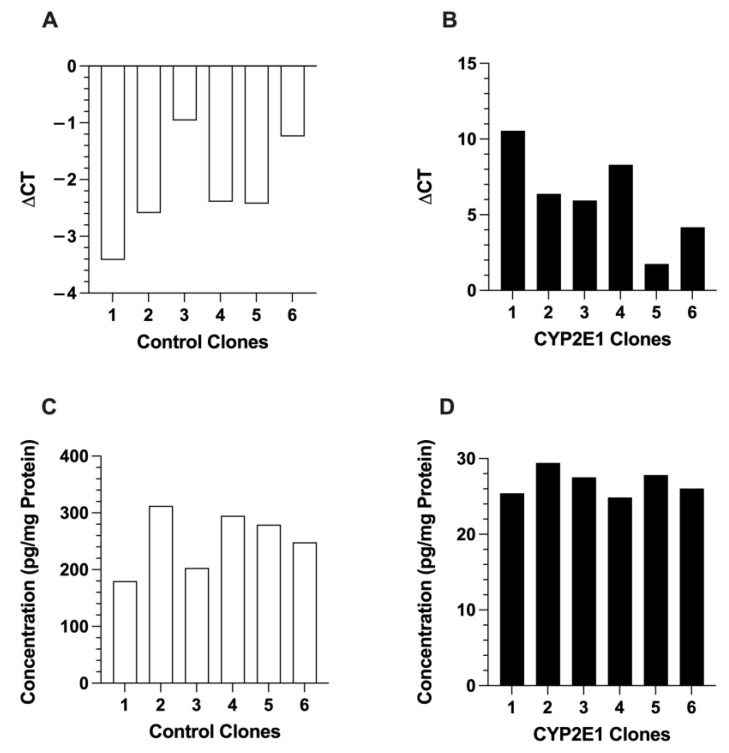
The mRNA expressions (**A**,**B**) and protein contents (**C**,**D**) of GFP in the six control (**A**,**C**) and six CYP2E1 (**B**,**D**) clones of stably transfected HepG2 cells. Each column represents the average of two replicates.

We further confirmed the expression and its relative quantitative abundance by the mRNA expression and protein contents of GFP in the six control and six CYP2E1 clones. As shown in Figure 2, the expressions of GFP in the control clones (Figure 2A) were much higher than those in the CYP2E1 clones (Figure 2B), as evidenced by the lower ΔCT values due to higher GFP mRNA transcripts, resulting in early amplification in RT-PCR. The ΔCT values ranged from −0.94 (clone 3) to −3.4 (clone 1) for the control clones (Figure 2A) but ranged from 1.8 (clone 5) to 11 (clone 1) for the CYP2E1 clones (Figure 2B). In general agreement with the mRNA data, the GFP protein levels, measured by ELISA, in the control clones (Figure 2C) were around 10-fold higher than those in the CYP2E1 clones (Figure 2D). 

**Figure 3:**
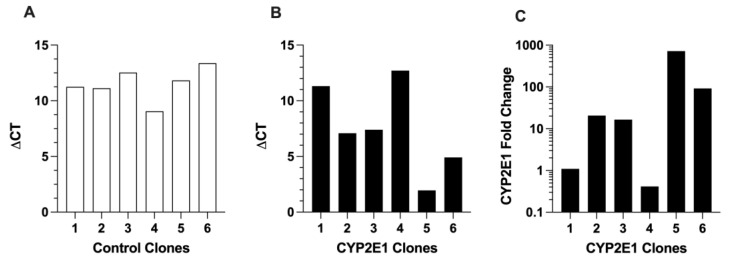
The mRNA expressions of CYP2E1 in the six control (**A**) and six CYP2E1 (**B**) clones of stably transfected HepG2 cells, and the fold change in the mRNA expression of CYP2E1 in the CYP2E1 clones relative to the geometric mean of the expressions in the control clones (**C**). Each column represents the average of two replicates.

The CYP2E1 expressions in the control and CYP2E1 clones are presented in Figure 3. The ΔCT values for CYP2E1 expression in the control clones (Figure 3A), which are indicators of very low expression of wild-type CYP2E1, were in the very high range of 9.1 (clone 4) and 13 (clone 6). The ΔCT values for the CYP2E1 clones (Figure 3B) were between 2.0 (clone 5) and 13 (clone 4). The fold increases in the CYP2E1 expression, calculated by the ΔΔCT method, are shown in Figure 3C. Based on these data, clone 5 had the highest overexpression of CYP2E1, as it showed a 720-fold higher CYP2E1 expression compared with the geometric mean expression of CYP2E1 in the control clones (Figure 3C). The high mRNA overexpression of CYP2E1 in clone 5 (Figure 3B,C) was in agreement with the high mRNA expressions of GFP (Figure 2B) and GFP fluorescence (Figure 1A) in this clone, which were the highest among the CYP2E1 clones. 

**Figure 4:**
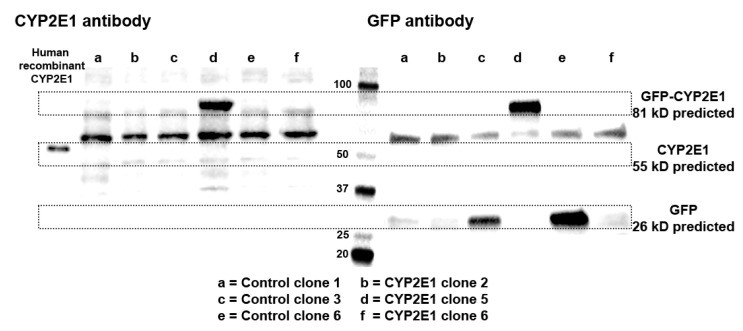
Western blot images of three CYP2E1 clones of stably transfected HepG2 cells with relatively high mRNA expression of CYP2E1 (Clones 2, 5, and 6), and three control clones of stably transfected HepG2 cells (clones 1, 3, and 6) using CYP2E1 (left panel), or GFP (right panel) antibodies. For comparison, the band for human recombinant CYP2E1 is also shown with the CYP2E1 antibody (left panel).

Next, we further confirmed the expression of chimeric GFP-CYP2E1 expression in the HepG2 clones by western blot analysis using two complimentary approaches, i.e., by probing with CYP2E1 and GFP antibodies, since the chimeric protein should be detected by both antibodies and should have the same predicted molecular weight in both these blots. Figure 4 depicts the western blot images of three CYP2E1 clones with relatively high mRNA expression of CYP2E1 (clones 2, 5, and 6) and three control clones (clones 1, 3, and 6). When the blots were probed with the CYP2E1 antibody (Figure 4, left panel), neither the CYP2E1-overexpressing nor the control clones showed any visible band of wild-type CYP2E1 at the expected molecular weight of ~55 kD. On the other hand, as anticipated, CYP2E1 clone 5 with the higher mRNA expression of CYP2E1 indeed showed a band for the GFP-tagged CYP2E1 protein with an expected MW of ~81 kD (Figure 4. Left panel). Furthermore, the presence of the CYP2E1-GFP band in the CYP2E1 clone 5 was also confirmed by a GFP antibody (Figure 4, right panel). It should also be noted that the control clones also showed the anticipated GFP protein band with an expected MW of ~26 kD, with the order of intensities being clone 6 > clone 3 > clone 1 (Figure 4, right panel), which were qualitatively in agreement with the ELISA data (Figure 2C).

**Figure 5:**
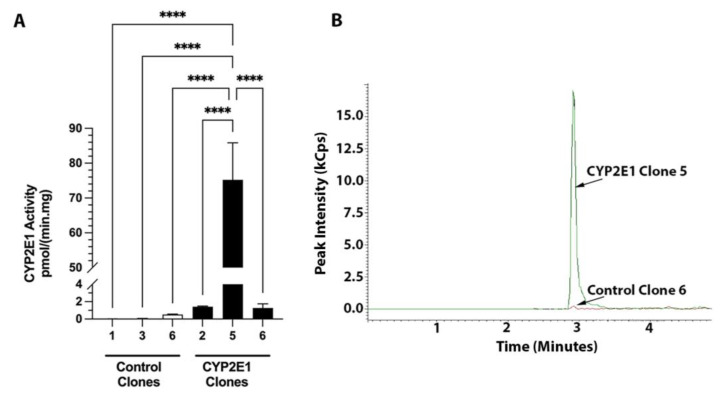
CYP2E1 activities of three control and three CYP2E1 clones of stably transfected HepG2 cells determined by the LC-MS/MS method (**A**) and the chromatograms of 6-hydroxychlorzoxazone metabolite formed by the microsomes of control (clone 6) and CYP2E1 (clone 5), which showed the highest CYP2E1 activities (**B**). The columns and bars, respectively, represent the mean and standard deviations of three replicate measurements. ****, *p* < 0.0001; based on the one-way ANOVA, followed by the Tukey’s multiple comparison test.

The CYP2E1 activities of the three control and three CYP2E1 clones determined by the LC-MS/MS method are presented in Figure 5A. Additionally, the representative chromatograms of the formed metabolite for the control and CYP2E1 clones with the highest activities are shown in Figure 5B. The chlorzoxazone hydroxylation activities of the three control clones were ≤0.5 pmol/(min·mg protein) (Figure 5A). However, the chlorzoxazone hydroxylation activity of the CYP2E1 clone 5 was 75 pmol/(min·mg protein) (Figure 5A), which is 150-fold more than the activity of the control clone 6 (0.5 pmol/min/mg), which had the highest activity among the control clones (Figure 5). 

### 2.2. Effects of CYP2E1 Overexpression on H/R-Induced Injury in HepG2 Cells

Based on the above characterization data, we selected the CYP2E1 clone 5 and control clone 6 to investigate the effects of CYP2E1 overexpression on the H/R injury using a model of 10 h hypoxia and 0.5 h of reperfusion. 

**Figure 6:**
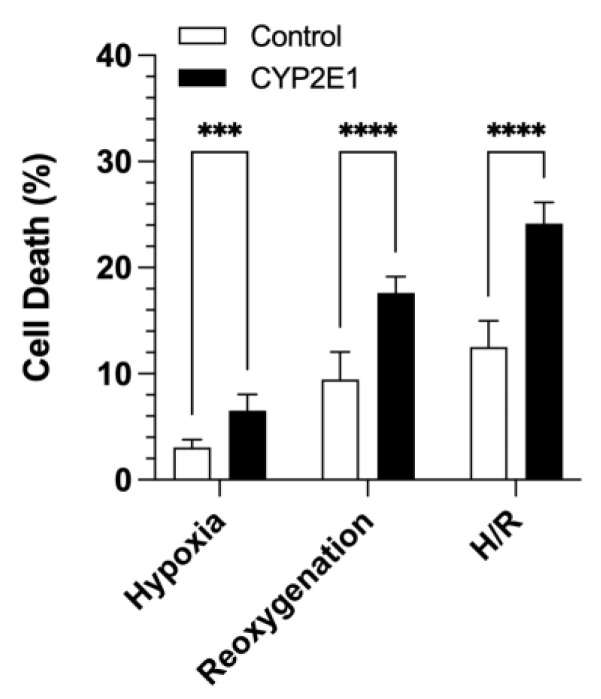
The effects of CYP2E1 overexpression on cell death during hypoxia, reoxygenation, and hypoxia/reoxygenation in the control and CYP2E1 clones. Cell death was quantitated by LDH release. The columns and bars, respectively, represent the mean and standard deviations of twelve measurements in three separate experiments. ***, *p* < 0.001; ****, *p* < 0.0001; based on the two-way ANOVA, followed by the Bonferroni’s multiple comparison test.

The effects of CYP2E1 overexpression on cell death due to H/R injury are presented in Figure 6. CYP2E1 overexpression significantly increased cell death during hypoxia (*p* < 0.001) and reoxygenation (*p* < 0.0001) by almost a factor of two (Figure 6). Similarly, the total cell death during combined hypoxia and reoxygenation increased from 12.5 ± 2.5% to 24.2 ± 2.0% in the CYP2E1-overexpressing cells compared to the control cells (*p* < 0.0001) (Figure 6). 

**Figure 7:**
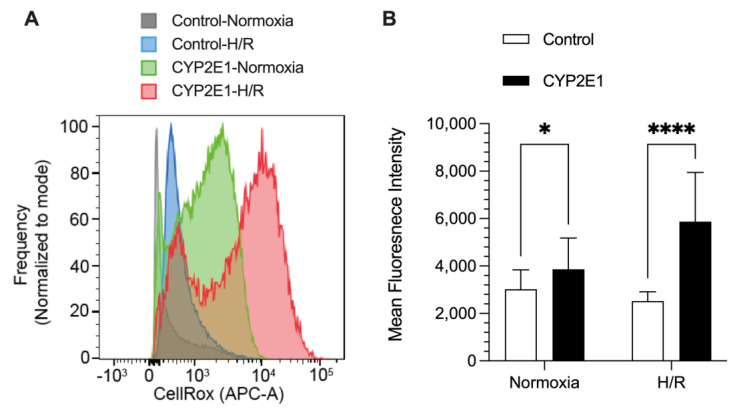
The effects of CYP2E1 overexpression on ROS generation determined by mean fluorescence intensity of CellROX deep red dye using flow cytometry after subjecting the control and CYP2E1 clones of stably transfected HepG2 cells to normoxia (basal) or hypoxia/reoxygenation (H/R). (**A**) Histograms for cell frequencies at each CellRox intensity for different conditions (indicated by histogram color) from a representative sample (**B**) the columns and bars, respectively, represent the mean and standard deviations of 25 measurements in 3 separate experiments. *, *p* < 0.05; ****, *p* < 0.0001; based on the two-way ANOVA, followed by the Bonferroni’s multiple comparison test.

The ROS generation, using CellROX^®^ Deep red dye, in the control and CYP2E1 HepG2 cells under both normoxia and H/R conditions are shown in Figure 7. Under normoxia (no H/R), the CYP2E1-overexpressed cells showed ~30% more ROS generation (*p* < 0.05) than the control cells. The ROS generated during the H/R condition was even more sensitive to CYP2E1 overexpression, as the CYP2E1 HepG2 cells produced 2.3-fold more ROS (*p* < 0.0001), compared with the ROS generated by the control cells (Figure 7).

## 3. Discussion

In this study, we developed and characterized a CYP2E1-overexpressing HepG2 cell line to investigate the role of CYP2E1 under pathophysiological conditions that cause liver injury. We selected the HepG2 cell line because it is a commonly used human hepatic cell line for pharmacological and hepatotoxicity studies [28]. Although HepG2 cells retain several physiological and metabolic functions of hepatocytes noted in the liver tissue, the levels of endogenous P450 enzymes are extremely low or undetectable in these cells [29]. However, HepG2 cells retain sufficiently high levels of NADPH-cytochrome P450 reductase (CPR) [30,31], which is the electron-donating system necessary for P450 function. The low P450 content, combined with the relatively high CPR activity of the HepG2 cells, is advantageous because the overexpression of individual P450 isoforms in these cells could be used to study the effects of the individual P450 isoforms on different pathophysiological or toxicological conditions in the absence of the confounding effects by other P450 isoforms. 

Based on the characterization studies, the highest overexpression of CYP2E1 in our studies was seen in CYP2E1 clone 5. The selection of CYP2E1 clone 5 was based on the following results. First, the percentage of the cells showing the GFP expression for CYP2E1 clone 5 (80.6 ± 4.3%) was the highest among all the six tested clones (Figure 1B). Second, CYP2E1 and GFP gene expression data showed that CYP2E1 clone 5 had the highest GFP (Δct = 1.8, Figure 2B) and CYP2E1 (Δct = 1.8, Figure 3B) gene expression, with a 720-fold CYP2E1 gene expression over the control clones (Figure 3C). Third, only CYP2E1 clone 5 showed prominent bands for GFP-tagged CYP2E1 protein with a molecular weight (MW) of ~81 kD in the western blot analysis of microsomal preparations using both CYP2E1 (Figure 4, left panel) and GFP (Figure 4, right panel) antibodies. Lastly, CYP2E1 overexpression in the HepG2 cell in clone 5 resulted in a 150-fold increase in the CYP2E1 activity in these cells (Figure 5). These data collectively indicate the successful generation of a CYP2E1-overexpressing HepG2 cell that could be used in future studies to investigate the role of CYP2E1 under various pathophysiological conditions. 

Both the mRNA and protein (ELISA) studies clearly showed that GFP expressions in the control clones were much higher than those in the CYP2E1 clones (Figure 2), an observation that was also in agreement with the markedly higher fluorescence intensities in the microscopic images of these clones (Figure 1). This difference could be related to the cytotoxic effect of CYP2E1 overexpression and ROS formation in the CYP2E1 clones, which leads to a slow growth rate and low cell viability in these cells as reported previously [32,33]. 

Cederbaum and colleagues previously generated a stable cell line constitutively expressing CYP2E1 by either the retroviral infection [34], or plasmid transfection [33] method. They have extensively used these cell lines to study the role of CYP2E1 in ROS generation and liver injury under different pathophysiological conditions, including ethanol toxicity [35]. Similar to our western blot results (Figure 4), this group showed that the control HepG2 cells do not show any detectable CYP2E1 bands [33]. However, the CYP2E1-overexpressed cells revealed prominent CYP2E1 bands. Additionally, whereas the control HepG2 cells had little or no CYP2E1 catalytic activity, the overexpressed HepG2 cells were catalytically active in metabolizing the CYP2E1 substrate p-nitrophenol [33], and several other CYP2E1 substrates such as dimethylnitrosamine, aniline, and ethanol [34]. Our catalytic activity data with chlorzoxazone are in agreement with the data reported by Cederbaum and colleagues. 

Previous in vivo studies from our laboratory [36,37,38] suggest that P450 enzymes may have a detrimental role in the hepatic I/R injury in animals. In a normothermic ischemia-reperfusion model in rats, diallyl sulfide, an inhibitor of CYP2E1, protected the liver from I/R injury by reducing oxidative stress [36]. Furthermore, in another study [37], the pretreatment of animals with the P450 inhibitor cimetidine completely or partially reversed all the I/R-mediated changes. The protective effects of cimetidine were associated with a 60% decline in the microsomal CYP2C11 activity [37]. Conversely, pretreatment of rats with the P450 enzyme inducer phenobarbital, exacerbated the hepatic IR injury [38]. Phenobarbital pretreatment caused an approximately 40% increase in the total P450 content of the liver, which was also associated with a 75% increase in ROS generation in the I/R group. Collectively, these in vivo data suggest that P450 enzymes, including CYP2E1, may play a negative role in the hepatic I/R injury, perhaps through ROS generation. However, as the inhibitors or inducers of P450 enzymes most likely exert other effects, the direct effects of P450 on the I/R injury cannot be unequivocally determined from these in vivo studies. 

In the current study, we overexpressed CYP2E1 in a HepG2 cell that normally contains little or no CYP2E1 activity to directly test the effects of CYP2E1 on the H/R injury in an ex vivo model, which is devoid of all the complexities of the in vivo models. Our data clearly shows that CYP2E1 exacerbates H/R injury, as evidenced by a substantial increase (~2-fold) in the H/R-induced cell death (Figure 6). As demonstrated in Figure 6, the extent of cell death in both the control and CYP2E1 clones during the 0.5 h of reoxygenation was almost 3-fold higher than that after the 10 h of hypoxia. This phenomenon is in agreement with previous reports that cell death mainly occurs during the reoxygenation phase of H/R injury [39]. 

Our ROS data (Figure 7) suggest that the increased cell death observed in the CYP2E1 clone after H/R injury (Figure 6) is due to, at least in part, to an increased ROS generation in these cells. A 1.9-fold increase in cell death in the CYP2E1 clone (Figure 6) was matched by a 2.3-fold increase in ROS generation (Figure 7) under the H/R condition. Additionally, the normoxia data presented in Figure 7 shows that the CYP2E1 clone also generates ~30% more ROS (*p* < 0.05) than the control clone under the basal condition (Figure 7). This observation is in agreement with the previously reported studies that CYP2E1 plays a crucial role in oxidative stress via ROS generation [27,34,40]. 

A unique characteristic of CYP2E1, compared with other P450 enzymes, is that its heme iron is constitutively in the high spin state [41]. Due to its constitutive high spin, CYP2E1 generates ROS, such as superoxide and hydrogen peroxide, in the absence of substrates, and is therefore widely considered as a ‘leaky’ enzyme [41]. Additionally, the increase in the ROS generation by CYP2E1 under H/R could be potentially related to the CYP2E1-increased degradation and the release of heme and iron due to H/R, which may contribute to the generation of hydroxyl radicals in the Fenton reaction [15]. The latter postulate is in agreement with the findings that iron overload plays a novel role in hepatic I/R injury, and ferroptosis, which is an iron-dependent cell death, and is involved in the hepatic I/R injury pathogenesis [42]. Future studies are needed to distinguish between the contribution of the ROS generated from the catalytic cycle of CYP2E1 and that produced from the CYP2E1 degradation and generation of iron.

It has been suggested that the use of antioxidants or mechanisms to change the balance of the cellular oxidative stress antioxidants system in favor of antioxidant mechanisms could be a therapeutic strategy to mitigate hepatic I/R injury [11]. These strategies may include the exogenous administration of antioxidants such as N-acetylcysteine, superoxide dismutases, α-tocopherol, or herbal antioxidants [11]. If future studies confirm the role of CYP2E1 as a significant generator of ROS in hepatic I/R injury, therapeutic interventions may be directed at reducing ROS generation by CYP2E1, which may include strategies to inhibit or stabilize the enzyme during the surgical procedure.

## 4. Materials and Methods

### 4.1. Chemicals and Reagents

The cDNA for human cytochrome P450 2E1 (CYP2E1) with a C terminus-tagged turbo green fluorescent protein (tGFP), and its corresponding pCMV6-AC-GFP mammalian expression vector (control) were purchased from OriGene Technologies (Rockville, MD, USA). Lipofectamine 2000, Superscript III First Strand kit (synthesis system for RT-PCR), CyQUANT™ LDH cytotoxicity assay kit, and CellROX^®^ Deep Red Reagent kit were all obtained from Invitrogen (Carlsbad, CA, USA). Eagle’s minimum essential medium (EMEM) and HepG2 cells were purchased from ATCC (Manassas, VA, USA). Gibco Geneticin (G418), fetal bovine serum (FBS), and Pierce RIPA buffer, containing protease inhibitors, were obtained from Thermo Scientific (Rockford, IL, USA). The RNA extraction kit (RNeasy) and the GFP ELISA kit were purchased from Qiagen Inc. (Valencia, CA, USA) and Cell Biolabs, Inc. (San Diego, CA, USA), respectively. The primary antibodies against human CYP2E1 (ab28146), and the secondary antibody (Ab6721) were purchased from Abcam (Cambridge, MA, USA). Mouse monoclonal turboGFP antibody was purchased from OriGene Technologies (Rockville, MD, USA). HRP-conjugated goat anti-mouse IgG (H+L) secondary antibody for GFP detection was obtained from Invitrogen (Carlsbad, CA, USA). Chlorzoxazone, 6-hydroxychlorzoxazone, NADPH, and 4-methylpyrazole (4-MP) were bought from Sigma-Aldrich (St. Louis, MO, USA). 6-Hydroxychlorzoxazone-d2 was procured from Santa Cruz Biotechnology (Santa Cruz, CA, USA). All the other chemicals and reagents were of high purity and purchased from commercial sources. 

### 4.2. Cell Line and Culture Conditions 

HepG2 cells were grown in EMEM media that contained 1% penicillin–streptomycin antibiotic mixture and 10% FBS. The culture was kept at 37 °C in the incubator with 5% CO_2_ and 95% air atmosphere. 

### 4.3. Generation of HepG2 Cells with Stable Overexpression of CYP2E1

The transfection was carried out according to the ATCC protocol (TransfeX™ Transfection of Plasmid DNA into HepG2 Cells) with minor modifications. HepG2 cells were transfected with human CYP2E1 (tGFP-tagged) expressing plasmid CMV6 entry vector or its corresponding tGFP-tagged control vector (pCMV6-AC-GFP). The transfection was performed when the cells became 50% to 70% confluent (within 24 h), using Lipofectamine 2000 at a ratio of ~2 µg:5 µL/well (DNA: Lipofectamine). Briefly, HepG2 cells were seeded into a six-well plate (70 × 10^4^ cells/well). After overnight culture, the complete media was replaced with Opti-MEM reduced serum medium. To prepare the DNA-lipofectamine complex, 300 µL of Opti-MEM reduced serum medium containing 12 µL of lipofectamine 2000 was added to 300 µL of the medium containing 5 µg of DNA. The DNA–lipofectamine mixture was incubated for 15 min at room temperature to form the complex. Finally, 250 µL of the DNA–lipofectamine mixture was diluted to 1 mL with Opti-MEM reduced serum medium and added to the well containing the cells. The next day, the transfection complex-containing media was replaced with the EMEM media containing 10% FBS and 1400 µg/mL of G418 for stable selection. The GFP-positive cells were observed under the fluorescence microscope until G418-resistant scattered colonies were formed around day 15. Glass rings were used to select and isolate six clones from each transfection group (CYP2E1 and the control), grown to large scale, and kept in EMEM media containing 10% FBS, 200 µg/mL G418, and 2 mM 4-methylpyrazole (4-MP) to stabilize the CYP2E1 protein [34]. After subjecting the six clones in each group to the characterization steps described in subsequent sections, one clone from each group was selected for in vitro hypoxia-reperfusion (H/R) studies described below.

### 4.4. Quantification of CYP2E1 and GFP Gene Expression

The mRNA was isolated from CYP2E1 and control stable clones of HepG2 cells using the Qiagen RNeasy kit, following the manufacturer’s protocol. The mRNA was immediately reverse transcribed to cDNA using the Superscript III First-Strand Synthesis kit for complementary cDNA synthesis. The cDNA was used to quantify CYP2E1 and GFP gene expressions, and β-actin was used as the reference gene using real-time PCR. Briefly, a reaction mixture was prepared by combining 2 μL of cDNA, 2 μL of forward primer (200 nM), 2 μL of reverse primer (200 nM), 4 μL of UltraPure™ DEPC-treated water, and 10 μL of 2× SYBR^®^ Green SuperMix to have a total volume of 20 μL. In a thermocycler (Biorad CFX thermocycler; Bio-Rad Laboratories, Hercules, CA, USA), the mixture was run at a universal cycle, which included incubation at 95 °C for 10 min, followed by 40 cycles at 95 °C for 15 s, and 55 °C for 60 s. The gene expression of GFP or CYP2E1 was calculated using the ΔCT method:

ΔCT (GFP or CYP2E1) = CT (GFP or CYP2E1) − CT(β-actin)

where CT refers to the threshold cycle. The fold change in the CYP2E1 expression in the CYP2E1-overexpressed cells relative to the wild-type CYP2E1 expression was estimated by the ΔΔCT method:

ΔΔCT (CYP2E1) = ΔCT (CYP2E1 Clone) − ΔCT (Control Clone)

Fold Change = 2^−ΔΔCT (CYP2E1)^

where ΔCT (Control Clone) is the geometric average of CYP2E1 ΔCT for all 6 control clones.

### 4.5. ELISA Quantification of GFP

The GFP protein levels were quantified in the CYP2E1 and control stable clones of HepG2 cells using a commercially available ELISA kit after preparing the cell lysates using RIPA buffer that contained a protease inhibitor cocktail. The total protein in the clone lysates was quantified by the bicinchoninic acid (BCA) protein assay, using bovine serum albumin as standard, and the GFP protein levels were normalized for the total protein in the cell lysates.

### 4.6. Microsomal Preparation

Stably transfected HepG2 clones were grown until confluence in 2 T-75 flasks (~20 million cells). The microsomes were prepared by the differential centrifugation method [43]. Briefly, after removing the media, the cells in each flask were harvested by scraping and then suspended in 10 mL of 100 mM of potassium phosphate buffer (pH 7.4). The cells were then homogenized on ice using an ultrasonic probe for 15 s (pulse on 01 s, pulse off 01 s, and Amplitude 50%). The homogenate was subjected to centrifugation at 10,000 *g* for 15 min, followed by the ultracentrifugation of the supernatant at 300,000 *g* for 45 min. The resultant microsomal pellets were resuspended in 150 µL of 100 mM potassium phosphate buffer (pH 7.4). The protein concentration of the microsomes was quantified using a Quick Start™ Bradford Protein Assay (Bio-Rad; Hercules, CA, USA) using bovine serum albumin as standard.

### 4.7. Western Blot Analysis

CYP2E1 and GFP protein expression in the CYP2E1 and control stable clones of HepG2 cells were confirmed using western blot analysis of the microsomes. Briefly, 25 µg microsomes prepared from CYP2E1 and GFP clones of HepG2 cells was resolved by 4–20% sodium dodecyl sulfate–polyacrylamide gel electrophoresis on a precast Bio-Rad gel (Hercules, CA, USA), and then transferred to a polyvinylidene fluoride membrane. The membranes were blocked with 5% (*w*/*v*) BSA for 1 h at room temperature. Subsequently, the membranes were probed overnight (4 °C) with rabbit CYP2E1 primary antibody (1:2000 dilution, Abcam, ab28146) or mouse anti-GFP (1:1000 dilution, OriGene, TA150041). To detect CYP2E1 protein, the membrane was then incubated with horseradish peroxidase (HRP)-conjugated goat anti-rabbit secondary antibody (1:5000 dilution, Ab6721) and StrepTactin-HRP conjugate for 1 h at room temperature. To detect GFP protein, the membrane was incubated with HRP-conjugated goat anti-mouse IgG (H+L) secondary antibody (1:2500 dilution, Invitrogen, 62-6520) and StrepTactin-HRP conjugate for 1 h at room temperature. The bands were detected using the Bio-Rad ChemiDoc Imaging system.

### 4.8. CYP2E1 Activity

The CYP2E1 activity was measured in the microsomal fractions based on the formation of 6-hydroxychlorzoxazone from the CYP2E1 substrate chlorzoxazone [44]. The incubation procedure and analytical method for the quantitation of the metabolite were similar to those reported previously [45], with some modifications. Briefly, reaction mixtures (50 µL in pH 7.4 Tris-HCl buffer) containing the microsomes (500 µg/mL) and chlorzoxazone (200 µM) were preincubated for 5 min at 37 °C before initiating the reaction with NADPH (1 mM). After 20 min incubation at 37 °C, the reaction was stopped by adding 150 µL of ice-cold acetonitrile, containing 20 nM deuterated metabolite (6-hydroxychlorzoxazone-d2) as the internal standard. The samples were vortexed, kept on ice for 5 min, and centrifuged at 13,400 rpm for 5 min. The supernatants were transferred to the autosampler vials and injected onto the LC-MS/MS system (EVOQ Elite UPLC-MS/MS; Bruker Scientific LLC, Billerica, MA, USA) with a Kinetex 1.7 μm C18 column (50 × 2.1 mm, 100 A) from Phenomenex Inc Torrance, CA, USA). The 6-hydroxychlorzoxazone (metabolite) concentrations were analyzed using a gradient mobile phase, consisting of mobile phase A (5 mM ammonium formate and 0.05% formic acid in water), and B (acetonitrile: methanol: formic acid; 95:5:0.05, *v*/*v*). The gradient mobile phase (0–0.8 min, 10% B; 0.8–4.5 min, linear gradient 10–95% B; 4.5–6.5 min, 95% B; 6.5–7 min, 10% B; 7–8.5 min, 10% B) was delivered at a flow rate of 0.2 mL/min. A calibration standard ranging from 1–1000 nM of 6-hydroxychlorzoxazone was used to quantify the metabolite concentrations. All reactions were carried out in triplicate.

### 4.9. In Vitro Hypoxia/Reperfusion Model

Based on the characterization experiments, CYP2E1 clone 5 and control clone 6 of HepG2 cells were selected for the H/R studies. The H/R model, which uses anaerobic KRH buffer pH 6.2 during hypoxia, and aerobic KRH buffer pH 7.4 during reperfusion, was similar to those reported before [46,47,48]. Briefly, 20 × 10^4^ cells/well were seeded in a 24-well plate with EMEM media containing 10% FBS, 200 µg/mL G418, and 2 mM 4-MP, and were incubated for 48 h in the incubator with 5% CO_2_ and 95% air atmosphere. After that, the media was replaced with KRH buffer pH 6.2, and the plate was placed in a hypoxic chamber (MIC-101; Billups-Rothenberg; San Diego, CA, USA). The chamber was flushed with 95% nitrogen and 5% carbon dioxide to achieve hypoxia, and incubated for 10 h at 37 °C. At the end of hypoxia, the KRH pH 6.2 was replaced with the oxygenated KRH pH 7.4 and subjected to reoxygenation for 30 min at 37 °C in the incubator with 5% CO_2_ and 95% air atmosphere. Finally, as described below, the cells were used to assess H/R injury markers, such as cell viability and ROS generation. 

### 4.10. Quantitation of Cell Viability Using LDH Assay

At the end of 10 h of hypoxia and after 30 min of reoxygenation, the KRH 6.2 and KRH 7.4 media were collected to measure the released LDH during the hypoxia and reperfusion, respectively. Furthermore, after removal of the reperfusion media, the cells were permeabilized with 375 µmol/L digitonin (final concentration in KRH 7.4) to determine the total LDH release that was associated with 100% cell death [46]. The concentrations of LDH in the media were quantitated using the CyQUANT™ LDH Cytotoxicity Assay Kit (Invitrogen), following the manufacturer’s protocol. The analysis was done using a microplate reader (SpectraMax^®^ M5; Molecular Devices; San Jose, CA, USA). The experiments were done in quadruplicate and repeated three times (*n* = 12). 

The cell death percentages during hypoxia ($\%Cell\;{Death}_{H}$), reperfusion ($\%Cell\;{Death}_{R}$), and hypoxia/reperfusion ($\%Cell\;{Death}_{H/R}$) were calculated as shown below:

$$\%Cell\;{Death}_{H}=\frac{{LDH}_{H}}{{LDH}_{Total}}\times 100$$

$$\%Cell\;{Death}_{R}=\frac{{LDH}_{R}}{{LDH}_{Total}}$$

$$\%Cell\;{Death}_{H/R}=\%Cell\;{Death}_{H}+\%Cell\;{Death}_{R}$$

where *LDH_H_*, *LDH_R_*, and *LDH_Total_* are the concentrations of *LDH* in the media at the end of hypoxia, reperfusion, and after the addition of digitonin, respectively.

### 4.11. ROS Generation

To determine the effect of CYP2E1 overexpression on ROS generation under H/R, CellROX^®^ Deep Red Reagent was used following the manufacturer’s protocol. Briefly, at the end of hypoxia, KRH pH 6.2 was replaced with oxygenated KRH pH 7.4, containing 5 µM CellROX^®^ Deep Red dye, and the cells were incubated for 30 min at 37 °C to mimic reperfusion. Subsequently, the cells were washed with PBS and harvested into a 96-well round-bottom plate pelleted at 1500 rpm for 5 min. The cells were then resuspended in 200 µL PBS containing viability stain (BD 564406) and incubated for 10 min at room temperature. Cells were washed once with PBS and resuspended in 200 µL PBS for flow cytometry analysis. The effect of CYP2E1 overexpression on ROS generation under basal conditions (normoxia) was also tested. Briefly, the cells were incubated with the complete media at 37 °C in a cell culture incubator with 5% CO_2_ and 95% air (normoxia) for 10 h to mimic the hypoxia duration. Subsequently, the media was replaced with a complete media, containing 5 µM CellROX^®^ Deep Red dye, and the cells were incubated for an additional 30 min at 37 °C. The cells were then prepared for flow cytometry analysis as described above. The flow cytometry analysis was conducted using BD LSRFortessa™ X-20 Cell Analyzer (BD Biosciences; San Jose, CA, USA), with at least 100,000 events acquired for each experimental condition and replicate. FlowJo^TM^ software (BD Biosciences) was used to analyze the flow cytometry data. The experiments were carried out over three separate days with n of 3, 8, and 14, for a total n of 25 for each group. Negative and positive controls were also examined in the absence of the dye and the presence of tert-butyl hydroperoxide, respectively. 

### 4.12. Statistical Analysis

The statistical analysis of data was carried out using GraphPad Prism software (La Jolla, CA, USA). The statistical differences among the different control and CYP2E1 clones in their CYP2E1 activities were analyzed using the one-way ANOVA, followed by the Tukey’s multiple comparison test. The comparison of %cell death or ROS generation between the selected CYP2E1 and control clones was conducted using the two-way ANOVA, followed by the Bonferroni’s multiple comparison test. The two factors were clone types and oxygenation status. All results are presented as mean ± SD.

## 5. Conclusions

In this study, stable CYP2E1-overexpressing HepG2 cells and GFP control cells were developed. Gene expression, CYP2E1 and GFP protein content, and CYP2E1 activity confirmed that CYP2E1 and GFP-control genes were successfully transfected permanently. The role of CYP2E1 in an ex vivo model of H/R injury was investigated by exposure of the CYP2E1 and control HepG2 cells to H/R, and quantitation of ROS generation and cell death. The results show that CYP2E1 significantly increases H/R-induced cell death and ROS production. Overall, these ex vivo studies suggest that the constitutive CYP2E1 present in the liver may exacerbate ROS generation and injury during hepatic ischemia-reperfusion injury in humans. However, further studies are necessary to understand the exact mechanisms by which CYP2E1 causes the detrimental increase in ROS generation after H/R injury. By understanding the specific role that CYP2E1 plays in the exacerbation of oxidative stress and inflammation following hepatic I/R, more refined therapeutic strategies may be developed.

## Data Availability

The data that support the findings of this study are available upon request from the corresponding authors.

## References

[1] Dar W.A., Sullivan E., Bynon J.S., Eltzschig H., Ju C. (2019). Ischemia reperfusion injury in liver transplantation: Cellular and molecular mechanisms. Liver Int..

[2] Jaeschke H., Lemasters J.J. (2003). Apoptosis versus oncotic necrosis in hepatic ischemia/reperfusion injury. Gastroenterology.

[3] Jaeschke H., Farhood A. (1991). Neutrophil and Kupffer cell-induced oxidant stress and ischemia-reperfusion injury in rat liver. Am. J. Physiol..

[4] Zhang W., Wang M., Xie H.Y., Zhou L., Meng X.Q., Shi J., Zheng S. (2007). Role of reactive oxygen species in mediating hepatic ischemia-reperfusion injury and its therapeutic applications in liver transplantation. Transpl. Proc..

[5] Granger D.N., Kubes P. (1994). The microcirculation and inflammation: Modulation of leukocyte-endothelial cell adhesion. J. Leukoc. Biol..

[6] Zhai Y., Busuttil R.W., Kupiec-Weglinski J.W. (2011). Liver ischemia and reperfusion injury: New insights into mechanisms of innate-adaptive immune-mediated tissue inflammation. Am. J. Transpl..

[7] Abu-Amara M., Yang S.Y., Seifalian A., Davidson B., Fuller B. (2012). The nitric oxide pathway—Evidence and mechanisms for protection against liver ischaemia reperfusion injury. Liver Int..

[8] Jaeschke H. (2003). Molecular mechanisms of hepatic ischemia-reperfusion injury and preconditioning. Am. J. Physiol. Gastrointest. Liver Physiol..

[9] Lentsch A.B., Kato A., Yoshidome H., McMasters K.M., Edwards M.J. (2000). Inflammatory mechanisms and therapeutic strategies for warm hepatic ischemia/reperfusion injury. Hepatology.

[10] Yang W., Chen J., Meng Y., Chen Z., Yang J. (2018). Novel Targets for Treating Ischemia-Reperfusion Injury in the Liver. Int. J. Mol. Sci..

[11] Jaeschke H., Woolbright B.L. (2012). Current strategies to minimize hepatic ischemia-reperfusion injury by targeting reactive oxygen species. Transpl. Rev..

[12] Zangar R.C., Davydov D.R., Verma S. (2004). Mechanisms that regulate production of reactive oxygen species by cytochrome P450. Toxicol. Appl. Pharmacol..

[13] Puntarulo S., Cederbaum A.I. (1998). Production of reactive oxygen species by microsomes enriched in specific human cytochrome P450 enzymes. Free Radic. Biol. Med..

[14] Ishihara Y., Sekine M., Nakazawa M., Shimamoto N. (2009). Suppression of myocardial ischemia-reperfusion injury by inhibitors of cytochrome P450 in rats. Eur. J. Pharm..

[15] Paller M.S., Jacob H.S. (1994). Cytochrome P-450 mediates tissue-damaging hydroxyl radical formation during reoxygenation of the kidney. Proc. Natl. Acad. Sci. USA.

[16] Yu J., Zhu H., Kindy M.S., Taheri S. (2021). Cytochrome P450 CYP2E1 suppression ameliorates cerebral ischemia reperfusion injury. Antioxidant.

[17] Guengerich F.P. (2022). Roles of cytochrome P450 enzymes in pharmacology and toxicology: Past, present, and future. Adv. Pharm..

[18] Cederbaum A.I. (2014). Molecular mechanisms of the microsomal mixed function oxidases and biological and pathological implications. Redox Biol..

[19] Zanger U.M., Schwab M. (2013). Cytochrome P450 enzymes in drug metabolism: Regulation of gene expression, enzyme activities, and impact of genetic variation. Pharmacol. Ther..

[20] Rashba-Step J., Cederbaum A.I. (1994). Generation of reactive oxygen intermediates by human liver microsomes in the presence of NADPH or NADH. Mol. Pharm..

[21] Ekstrom G., Ingelman-Sundberg M. (1989). Rat liver microsomal NADPH-supported oxidase activity and lipid peroxidation dependent on ethanol-inducible cytochrome P-450 (P-450IIE1). Biochem. Pharm..

[22] Ogaki S., Taguchi K., Watanabe H., Ishima Y., Otagiri M., Maruyama T. (2014). Carbon monoxide-bound red blood cell resuscitation ameliorates hepatic injury induced by massive hemorrhage and red blood cell resuscitation via hepatic cytochrome P450 protection in hemorrhagic shock rats. J. Pharm. Sci..

[23] Meyer U.A. (2007). Endo-xenobiotic crosstalk and the regulation of cytochromes P450. Drug Metab. Rev..

[24] Lindstrom T.D., Hanssen B.R., Bendele A.M. (1990). Effects of hepatic ischemia-reperfusion injury on the hepatic mixed function oxidase system in rats. Mol. Pharm..

[25] Pahan K., Smith B.T., Singh A.K., Singh I. (1997). Cytochrome P-450 2E1 in rat liver peroxisomes: Downregulation by ischemia/reperfusion-induced oxidative stress. Free Radic. Biol. Med..

[26] Caro A.A., Cederbaum A.I. (2004). Oxidative stress, toxicology, and pharmacology of CYP2E1. Annu. Rev. Pharm. Toxicol..

[27] Zhukov A., Ingelman-Sundberg M. (1999). Relationship between cytochrome P450 catalytic cycling and stability: Fast degradation of ethanol-inducible cytochrome P450 2E1 (CYP2E1) in hepatoma cells is abolished by inactivation of its electron donor NADPH-cytochrome P450 reductase. Biochem. J..

[28] Stanley L.A., Wolf C.R. (2022). Through a glass, darkly? HepaRG and HepG2 cells as models of human phase I drug metabolism. Drug Metab. Rev..

[29] Guo L., Dial S., Shi L., Branham W., Liu J., Fang J.L., Green B., Deng H., Kaput J., Ning B. (2011). Similarities and differences in the expression of drug metabolizing enzymes between human hepatic cell lines and primary human hepatocytes. Drug Metab. Dispos..

[30] Schulz C., Kammerer S., Kupper J.H. (2019). NADPH-cytochrome P450 reductase expression and enzymatic activity in primary-like human hepatocytes and HepG2 cells for in vitro biotransformation studies. Clin. Hemorheol. Microcirc..

[31] Waxman D.J., Lapenson D.P., Aoyama T., Gelboin H.V., Gonzalez F.J., Korzekwa K. (1991). Steroid hormone hydroxylase specificities of eleven cDNA-expressed human cytochrome P450s. Arch. Biochem. Biophys..

[32] Liu H., Lou G., Li C., Wang X., Cederbaum A.I., Gan L., Xie B. (2014). HBx Inhibits CYP2E1 Gene Expression via Downregulating HNF4α in Human Hepatoma Cells. PLoS ONE.

[33] Chen Q., Cederbaum A.I. (1998). Cytotoxicity and apoptosis produced by cytochrome P450 2E1 in Hep G2 cells. Mol. Pharm..

[34] Dai Y., Rashba-Step J., Cederbaum A.I. (1993). Stable expression of human cytochrome P4502E1 in HepG2 cells: Characterization of catalytic activities and production of reactive oxygen intermediates. Biochemistry.

[35] Wu D., Cederbaum A.I. (1996). Ethanol cytotoxicity to a transfected HepG2 cell line expressing human cytochrome P4502E1. J. Biol. Chem..

[36] Shaik I.H., George J.M., Thekkumkara T.J., Mehvar R. (2008). Protective effects of diallyl sulfide, a garlic constituent, on the warm hepatic ischemia-reperfusion injury in a rat model. Pharm. Res..

[37] Shaik I.H., Mehvar R. (2010). Effects of cytochrome P450 inhibition by cimetidine on the warm hepatic ischemia-reperfusion injury in rats. J. Surg. Res..

[38] Shaik I.H., Mehvar R. (2010). Cytochrome P450 induction by phenobarbital exacerbates warm hepatic ischemia-reperfusion injury in rat livers. Free Radic. Res..

[39] Qian T., Nieminen A.L., Herman B., Lemasters J.J. (1997). Mitochondrial permeability transition in pH-dependent reperfusion injury to rat hepatocytes. Am. J. Physiol..

[40] Gergel D., Misik V., Riesz P., Cederbaum A.I. (1997). Inhibition of rat and human cytochrome P4502E1 catalytic activity and reactive oxygen radical formation by nitric oxide. Arch. Biochem. Biophys..

[41] Harjumaki R., Pridgeon C.S., Ingelman-Sundberg M. (2021). CYP2E1 in lcoholic and non-alcoholic liver injury. Roles of ROS, reactive intermediates and lipid overload. Int. J. Mol. Sci..

[42] Yamada N., Karasawa T., Wakiya T., Sadatomo A., Ito H., Kamata R., Watanabe S., Komada T., Kimura H., Sanada Y. (2020). Iron overload as a risk factor for hepatic ischemia-reperfusion injury in liver transplantation: Potential role of ferroptosis. Am. J. Transpl..

[43] Cederbaum A.I. (2014). Methodology to assay CYP2E1 mixed function oxidase catalytic activity and its induction. Redox Biol..

[44] Shaik I.H., Mehvar R. (2011). Effects of normothermic hepatic ischemia-reperfusion injury on the in vivo, isolated perfused liver, and microsomal disposition of chlorzoxazone, a cytochrome P450 2E1 probe, in rats. J. Pharm. Sci..

[45] Chandrashekar D.V., DuBois B.N., Rashid M., Mehvar R. (2023). Effects of chronic cirrhosis induced by intraperitoneal thioacetamide injection on the protein content and Michaelis-Menten kinetics of cytochrome P450 enzymes in the rat liver microsomes. Basic Clin. Pharm. Toxicol..

[46] Pillai V.C., Snyder R.O., Gumaste U., Thekkumkara T.J., Mehvar R. (2011). Effects of transient overexpression or knockdown of cytochrome P450 reductase on reactive oxygen species generation and hypoxia-reoxygenation injury in liver cells. Clin. Exp. Pharm. Physiol..

[47] Pillai V.C., Mehvar R. (2011). Inhibition of NADPH-cytochrome P450 reductase by tannic acid in rat liver microsomes and primary hepatocytes: Methodological artifacts and application to ischemia-reperfusion injury. J. Pharm. Sci..

[48] Kim J.S., Ohshima S., Pediaditakis P., Lemasters J.J. (2004). Nitric oxide protects rat hepatocytes against reperfusion injury mediated by the mitochondrial permeability transition. Hepatology.

